# Timing of ripening initiation in grape berries and its relationship to seed content and pericarp auxin levels

**DOI:** 10.1186/s12870-015-0440-6

**Published:** 2015-02-12

**Authors:** Satyanarayana Gouthu, Laurent G Deluc

**Affiliations:** Department of Horticulture, College of Agricultural Sciences, Oregon State University, Corvallis, OR 97331 USA

**Keywords:** Seed, Auxin, Fruit ripening, *Vitis vinifera*, Asynchronous ripening

## Abstract

**Background:**

Individual berries in a grape (*Vitis vinifera* L.) cluster enter the ripening phase at different times leading to an asynchronous cluster in terms of ripening. The factors causing this variable ripening initiation among berries are not known. Because the influence *via* hormonal communication of the seed on fruit set and growth is well known across fruit species, differences in berry seed content and resultant quantitative or qualitative differences in the hormone signals to the pericarp likely influence the relative timing of ripening initiation among berries of the cluster.

**Results:**

At the time of the initiation of cluster ripening (véraison), underripe green berries have higher seed content compared to the riper berries and there is a negative correlation between the seed weight-to-berry weight ratio (SB) and the sugar level in berries of a cluster. Auxin levels in seeds relative to the pericarp tissues are two to 12 times higher at pre-ripening stages. The pericarp of berries with high-SB had higher auxin and lower abscisic acid (ABA) levels compared to those with low-SB from two weeks before véraison. In the prevéraison cluster, the expression of auxin-response factor genes was significantly higher in the pericarp of high-SB berries and remained higher until véraison compared to low-SB berries. The expression level of auxin-biosynthetic genes in the pericarp was the same between both berry groups based upon similar expression activity of *YUC* genes that are rate-limiting factors in auxin biosynthesis. On the other hand, in low-SB berries, the expression of ABA-biosynthetic and ABA-inducible *NCED* and *MYB* genes was higher even two weeks before véraison.

**Conclusions:**

Differences in the relative seed content among berries plays a major role in the timing of ripening initiation. Towards the end of berry maturation phase, low and high levels of auxin are observed in the pericarp of low- and high-SB berries, respectively. This results in higher auxin-signaling activity that lasts longer in the pericarp of high-SB berries. In contrast, in low-SB berries, concomitant with an earlier decrease of auxin level, the features of ripening initiation, such as increases in ABA and sugar accumulation begin earlier.

**Electronic supplementary material:**

The online version of this article (doi:10.1186/s12870-015-0440-6) contains supplementary material, which is available to authorized users.

## Background

Fruit set depends upon successful fertilization that includes regulatory interactions between fertilized ovule and ovary that are mediated by hormones [1]; fruit growth is also closely related to seed growth [2]. Seed number is positively correlated with fruit growth in many species including cucumber [3], grape [4], and sweet pepper [5]. For example, strawberry fruits fail to grow when the seeds are removed and growth can be restored upon the application of auxins to the deseeded fruits, indicating that seeds supply substances necessary for the fruit growth [6]. In grape, the weight of berries in the cluster may vary by a factor of two and the coefficient of variance of berry weight within a cluster can reach a maximum of 25-30% [7,8]. Many factors such as assimilate supply and environmental conditions may affect variation in berry size, but external factors such as water stress have been shown to homogeneously inhibit berry growth in all berries, indicating that internal factors influence berry growth differences [8]. The number of seeds has been suggested to influence cell division and cell expansion in pericarp through the production of hormones during tomato fruit development [1]. Similarly, lower growth was reported for seedless berries compared to seeded berries in Pinot Noir and Cabernet Sauvignon clusters [7]. Based upon recent molecular evidence, auxin is synthesized in the ovule and transported to the pericarp upon fertilization, where it induces gibberellin (GA) biosynthesis. The GA then degrades DELLA proteins that repress ovary growth and fruit initiation (reviewed by [9]). However, fruit development can be uncoupled from fertilization and seed development, as seen in parthenocarpic and stenospermocarpic fruits [10]. In these instances, elevated endogenous phytohormone levels in the pericarp similar to those of seeded fruits have been observed during fruit set [11]. This suggests that either the phytohormones could originate from sources other than seeds, or their increase in the pericarp is developmentally regulated. Accordingly, parthenocarpy can be induced by the exogenous application of auxins, cytokinins, or gibberellins [12] or by the expression of auxin biosynthetic genes in ovaries and ovules [13]. However, unseeded parthenocarpic fruits show less growth and lack the peak of auxin that occurs before the onset of ripening seen in the normal seeded fruits suggesting that, at least during the later stages of fruit development, the embryo-supplied auxin is necessary for continued fruit growth [14,15].

Even as we know much about the role of seeds during fruit set and maturation from several fruit models, the role played by seeds in the process of the ripening onset is not well understood. In strawberry, removal of achenes from immature fruit causes precocious ripening, which can be stopped by the application of auxin [16], suggesting that seeds negatively affect ripening through auxins. In tomato, over-expression of a gene from *Capsicum chinense* L. that encodes an auxin-conjugating enzyme (*GH3*) leads to increased sensitivity of fruit to ethylene at an earlier stage of development [17]. Similarly, in avocado, seeds have been shown to inhibit the ripening process, and seedless fruits show higher response to ethylene even at earlier developmental stages [18], probably owing to the lack of inhibitory action by the seed-supplied auxin. All of this evidence suggests that as seeds mature, auxin biosynthesis or transport to the pericarp ceases, allowing the mature fruit to ripen. This phenomenon appears to be supported across fruit species, including grape, as auxin treatment of fruit at immature and mature stages delays ripening [19-21]. It has been suggested that, as premature ripening of fruit before seed maturation is not desirable for reproductive success, seed and fruit maturation are strictly synchronized and auxin may coordinate communication between seed and pericarp [9].

In grape, completion of seed growth coincides with the onset of ripening (véraison) [22], when fruit becomes ready to undergo ripening and there is a major switch in the relative hormone levels in the pericarp, notably auxin and ABA [23,24]. In a mid-véraison cluster (50% of berries have changed color), uneven ripening among the individual berries indicates that the berries do not enter the ripening phase at the same time. Differences in flowering times and fertilization events have been suggested to cause the differences in the timing of ripening onset among berries [7]. However, Gorchov in [25] reported that variance in the fruit-ripening times in *Amelanchier arborea* appears more related to the developmental duration of fruit maturation rather than the flowering times. Based upon evidence for the seed-to-berry growth relationship discussed above, relative seed content in the berry might influence the duration of berry maturation and its transition to the ripening.

In the present study, we examined the differences in seed content between berries that enter the ripening stage sooner or later, with the aim of understanding the effect of seed content on the timing of berry ripening initiation. We monitored the changes in ripening-related hormones in the pericarp of berries with high or low seed content during the period leading to ripening onset. We show that the differences in the relative level of auxin in the berries having low and high seed content leads to differences in the timing of ripening initiation and could possibly be the main cause for asynchronous ripening of a grape cluster.

## Results and discussion

### Observation of seed content and berry ripening phenotype

**Figure 1:**
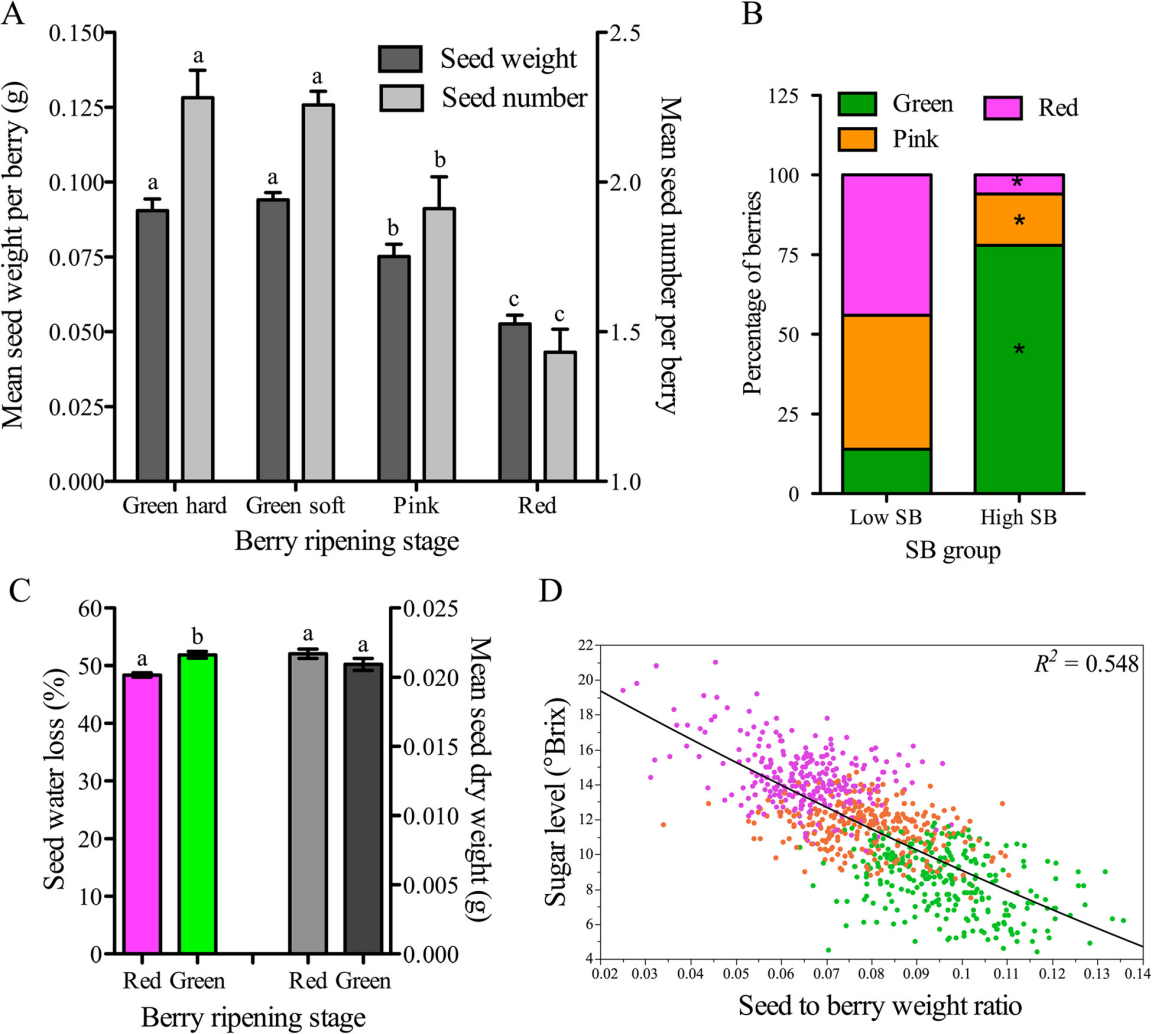
**Relationship between seed and berry ripening in the mid-véraison cluster. (A)** Seed weight and seed numbers in the green hard, green soft, pink, and red berries of mid-véraison cluster. Data represent means ± SEM (n = 50). Different letters denote significant differences between the berries of ripening stages for each seed parameter (Tukey’s HSD, *p* < 0.05) **(B)** Percentage of single-seeded green, pink and red berries in low and high seed weight-to-berry weight (SB) groups of berries in the cluster. Berries with low and high SB ranges were selected as described in methods. Berries of four entire clusters from two plants were used for analysis. Approximate test for equal proportions was used to identify differences in the distribution of ripening classes between the SB groups and significant differences were marked with asterisks (*p* < 0.05). **(C)** Differences in mean dry mass and dehydration levels per seed from green and red berries of mid-véraison cluster. Water loss was obtained by subtracting the seed dry weight from the fresh weight and expressed as percentage of water in the seed. Data represent means ± SEM (n = 50). Different letters denote significant differences between red and green berry seeds (Tukey’s HSD, *p* < 0.05). **(D)** Linear relation between SB and berry sugar level in the single-seeded berry population of a cluster. Sugar levels were measured as °Brix. Berries of ten clusters from five different plants were used for the analysis (n = 1,146).

Grape clusters generally transition into the ripening phase about eight weeks post-anthesis (E-L stage; [26]), but the timing of transition for individual berries in the cluster varies. Ripening-related physiological and transcriptional differences between berries in different ripening stages (green hard, green soft, pink soft, and red soft berries) in mid-véraison clusters have been reported in several studies [27-29]. In our previous study, by examining the post-véraison progression of sugar and color accumulation in differentially ripening berries within véraison clusters, we found that green hard, green soft, or pink berries were ~14, 7, or 4 days delayed, respectively, in their ripening program at mid-véraison stage (Additional file 1) [30]. Examination of the number and weight of seeds per berry revealed that green underripe berries consistently contained higher seed number and weight compared to the colored berries that had entered the ripening phase earlier even though the seeds had yet to reach their maximum weight (Figure 1A). This suggests that the delay in the ripening onset for individual berries increases along with the increasing seed content. A number of studies have indicated that seed number and weight are related to berry growth and showed a positive correlation between growth rates of seed and pericarp tissues during the first phase of berry development [4,7,22,31]. The entry of the pericarp into the ripening phase also generally coincides with the completion of seed growth [1]. Two studies investigated the relationship between seed content, fruit growth and ripening in grape [22,32]. In these studies lower seed weight in Shiraz and low seed number in Concord were associated with delayed berry ripeness and the ripening delay was attributed to incomplete seed maturation [22]. However, our results in Pinot noir show that green underripe berries have not only higher total seed weight per berry, but also the weights of individual seeds are significantly higher compared to those of red berries, suggesting a quantitative negative influence of seed tissue on the ripening (Additional file 2). These contrasting findings might be attributable to cultivar differences, to the method of defining berry seed content for which berry size differences were not considered, and to the exclusive use of seed weight or number in the interpretation of the results in the two studies cited in [22,32].

To examine the composition of green, pink, and red berries in the mid-véraison cluster originating from berries with low and high seed content, we segregated the berries on the basis of their “seed weight-to-berry weight ratio (SB)”, and refer to these as the low and high-SB groups hereafter. To rule out the effect of seed number on ripening, only the single-seeded berries, which accounted for ~70% of berries in a cluster, were considered (Figure 1B). While 86% berries with low-SB were either pink or red, 78% of high-SB berries were green at mid-véraison. This indicates that seed weight-to-berry weight ratio is a more suitable index to assess the influence of seed on the pericarp development than seed number per berry. The positive correlation between seed number and berry growth, reported in previous studies, was probably due to the fact that higher seed number generally results in higher weight. To examine whether low-SB in pink and red berries is due to dehydration of seeds, we compared the percentage of water loss between the seeds of green and red berries at mid-véraison (Figure 1C). Based upon the literature, dehydration begins in the middle of véraison [33]. In our data, seeds of red berries only had 3% less water content compared to those of green berries, indicating that they had just entered the dehydration phase. In addition, the dry mass of seeds was not different between green and red berries (Figure 1C) and the decrease in fresh weight did not commence before mid-véraison between low- and high-SB berries (Additional file 3). These results indicate that seed developmental changes in weight between green and red berries had little contribution to the observed SB ratio differences until mid-véraison. Further, SB showed a good correlation with sugar levels of the berries in the mid-véraison cluster, indicating that berries with low seed content begin to accumulate sugars, the main criterion for the ripening onset, earlier than those with high-SB content (Figure 1D).

### Ripening-related hormone levels in seed and pericarp tissues

**Figure 2:**
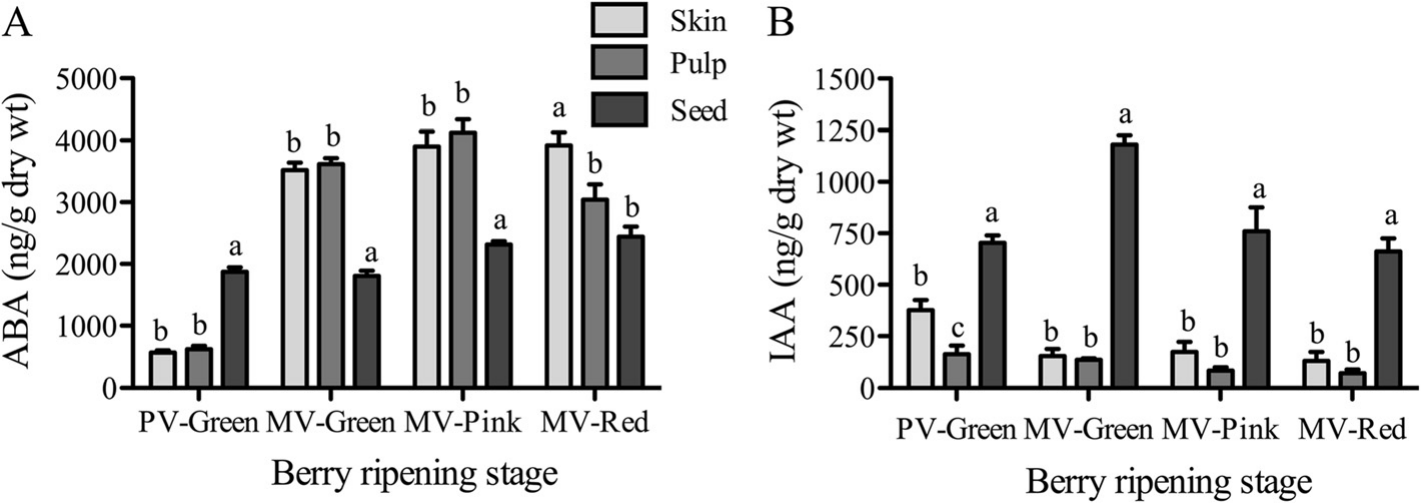
**Levels of ripening-related hormones (A) abscisic acid (ABA) and (B) indole-3-acetic acid (IAA) in skin, pulp, and seed tissues of berries.** Hormone levels were quantified in prevéraison green (PV) and green, pink, and red stage berries of a mid-véraison (MV) cluster. Error bars represent ± SEM (n = 4). Different letters denote significant differences between skin, pulp, and seed at each ripening stage (Tukey’s HSD, *p* < 0.05).

During the first cycle of rapid berry growth, the rate of seed growth is assumed to have a positive effect on the rate of cell division in the pericarp [2,34], which suggests that the seed supplies the pericarp with hormones required for cell division, and possibly cell expansion. In order to assess the relative levels of ripening-related hormones, we quantified abscisic acid (ABA) and indole-3-acetic acid (IAA), two main regulators of the ripening onset [20,24], in skin, pulp, and seed tissues of all green prevéraison clusters and the green, pink, and red berries from the mid-véraison-stage clusters (Figure 2). ABA levels in seeds were about 4 times higher than in pericarp tissues at prevéraison, but remained at a similar level until the later berry ripening-transition stage while ABA levels in the pericarp increased significantly during the berry ripening transitions (Figure 2A). ABA, a key regulator of seed maturation and embryogenesis, is largely synthesized in the integuments and its level in seeds is high during mid- and late-maturation stages (reviewed by [35]). Unlike in pericarp, where the actions of auxin and ABA are antagonistic and developmentally regulated during berry maturation and ripening phases, respectively, ABA and auxin act synergistically in seed to maintain dormancy [36]. In grape, rapid embryo growth and the associated synthesis of auxin occur around the maturation phase [22]. Auxin was up to 12-fold higher in the seeds during the pre-ripening phase and was maintained at significantly higher levels compared to pericarp tissues through the later ripening stages (Figure 2B). Pericarp tissues, especially pulp, show a steady decrease in IAA with progressing ripening stages. Evidence for seeds as the predominant source of auxins in fruit stems from studies of diverse species [23,37,38], in which synthesis of auxins in the embryo and endosperm tissues was observed [39]. Although mainly associated with normal embryo morphogenesis, auxin from seeds is thought to be transported to other parts of the fruit to promote cell division and expansion in the pericarp tissues [15,37,40]. When seed maturation is complete, auxin transport to the pericarp is inhibited, allowing the fruit to ripen (reviewed by [41]).

### Differences between berries with low and high seed weight-to-berry weight ratio (SB) within the prevéraison clusters

**Figure 3:**
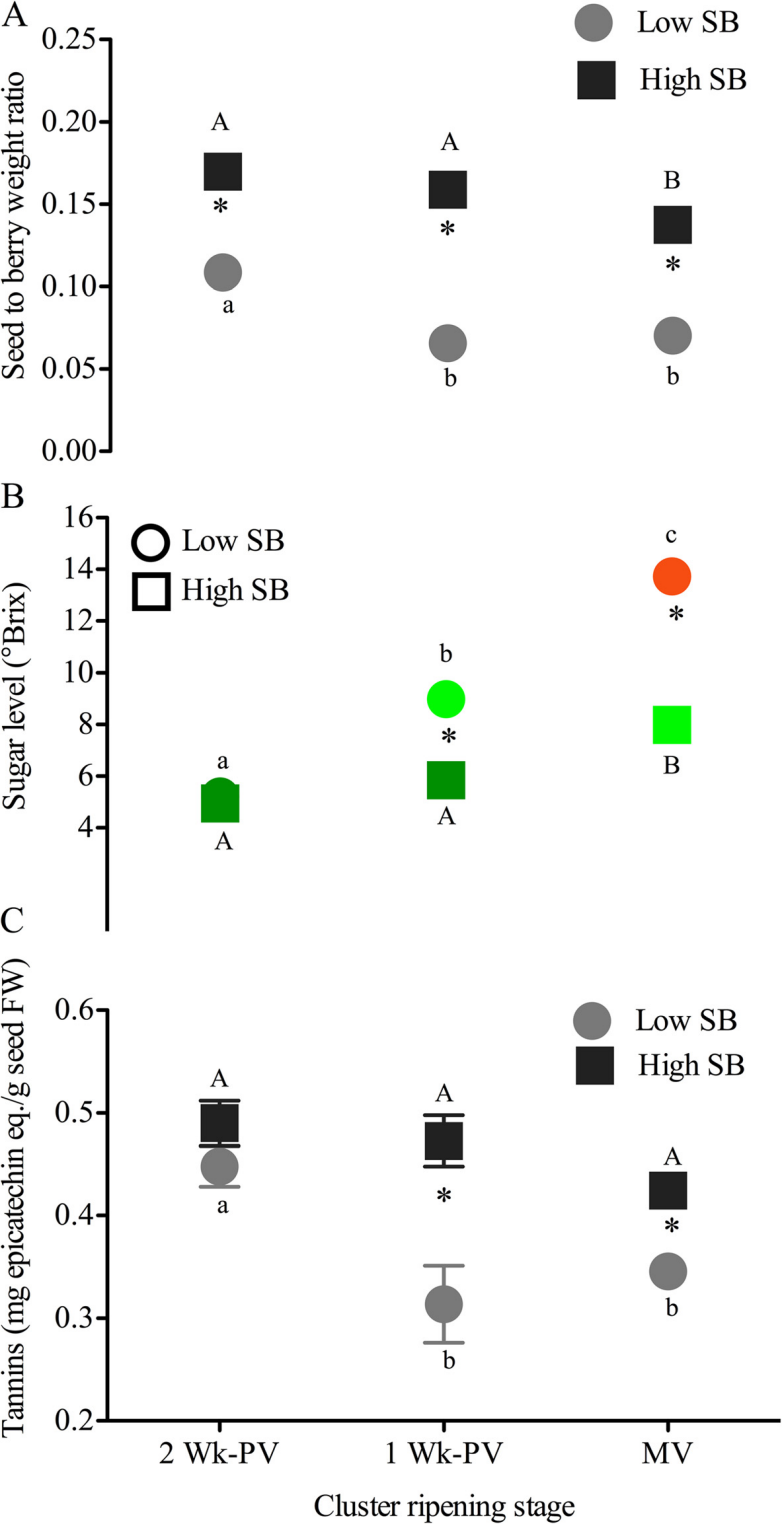
**Differences between low- and high-SB berry groups in (A) seed weight-to-berry weight ratio (SB), (B) sugar level and visual observation, and (C) seed tannin level at two- and one-week prevéraison, and at mid-véraison (MV) cluster stages.** The data represent the berries selected for downstream hormone and expression analyses based upon their highest SB differential. Error bars are ± SEM (n = 5). In panel **B**, the ripening phenotype of the berries of low and high-SB groups were indicated as dark green (green hard immature phase), light green (green soft phase with sugar increase), and red (red colored advanced ripening phase). The data representing low and high-SB berry populations are described in Additional file 3. Significant differences between low and high-SB at each cluster stage are indicated by asterisks (Student’s t-test, *p* < 0.05). Significant differences of each SB group between the ripening stages are denoted by different letters (lower and upper case letters are used for low and high-SB berries, respectively (Tukey’s HSD, *p* < 0.05)).

In most studies related to grape berry ripening, the definition of ripening progress has been based on overall cluster ripening and all green berries in prevéraison clusters were assumed to have low sugar, high auxin, and low ABA levels compared to those of a véraison-stage cluster [23,24,42]. However, given that the low- and high-SB berries of prevéraison clusters will emerge as different ripening classes (Figure 1B), the ripening-related biochemical and hormonal features might not be the same among all of the green prevéraison berries. Therefore, the pericarp of high- and low-SB berries from clusters that were at two or one week before mid-véraison (2-wk PV and 1-wk PV), or were at mid-véraison were analyzed separately. SB differences between low- and high-SB berries, selected for downstream hormone and gene expression analyses, were 1.6-, 2.4-, and 2-fold at 2-wk PV, 1-wk PV, and mid-véraison, respectively (Figure 3A). Prevéraison clusters that were two weeks before mid-véraison showed the highest correlation between seed and berry weights, followed by clusters at one week prevéraison and mid-véraison (data not shown). At 2-wk PV, all berries were at the end of the first rapid growth stage of the berry, when growth rates in seed and pericarp are most related [22], while at mid-véraison, the growth of the seeds concludes and the increase in pericarp weight is due to sugar import. The highest SB difference was observed at 1-wk PV and part of the rapid decrease in the ratio of the low-SB group can be attributed to pericarp expansion independent of seed influence (Additional file 3: Figures A and D). We observed the most rapid weight gain due to increases in sugars in high-SB berries after mid-véraison (Additional file 3: Figure D). At 2-wk PV, both low- and high-SB berries in the cluster were at the same pre-ripening stage based on similar basal sugar levels (Figure 3B). But one week later, the low-SB berries were already in the active sugar-accumulation phase, which indicates that these berries had entered the ripening transition earlier even though berries of both SB groups were still green (Figure 3B). In the mid-véraison cluster, low-SB berries were in pink to red stages with sugar levels at 14%, while high-SB berries were still at 8% (Figure 3B). In grape, seed tannins increase from very early stages of seed and berry development and reach a maximum around véraison, after which they rapidly decline [22,43]. So seed extractable tannins were used to assess the seed maturity differences between low and high-SB berries. Extractable tannins were at similar levels in the seeds of both low and high-SB berries in 2-wk PV clusters, but lower amounts of extractable tannins in the seeds of low-SB berries at 1-wk PV suggest that seeds were at an advanced developmental stage in these berries (Figure 3C). These results indicate that the developmental trajectory of the immature green berries deviate at least before one week prior to mid-véraison, depending on their seed content, and that the ripening-related differences would amplify further as the cluster approaches véraison-stage.

### Differences in auxin levels in the pericarp of low- and high-SB berries in clusters preceding the onset of ripening

**Figure 4:**
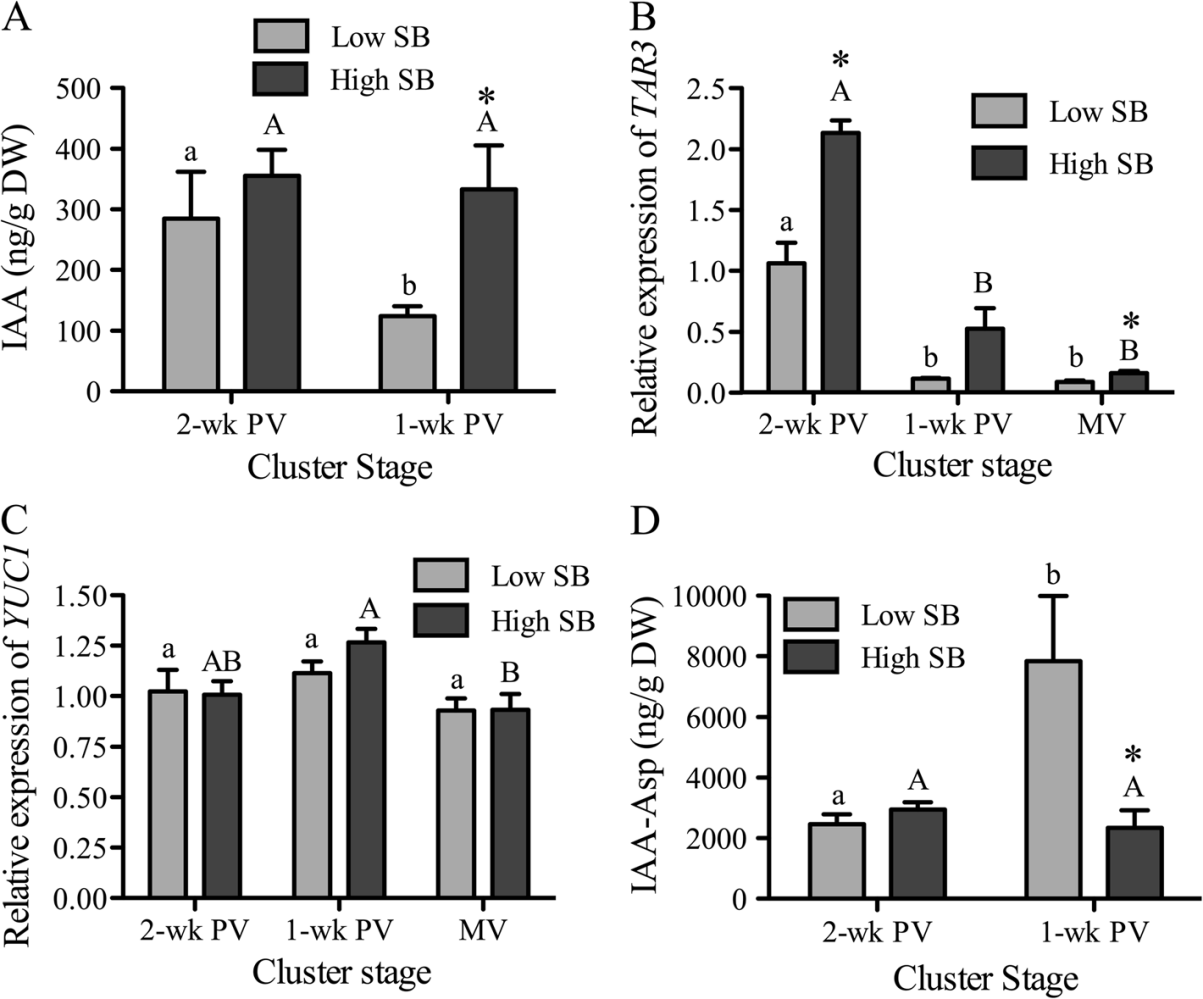
**Hormone levels of indole-3-acetic acid (IAA) (A) and its conjugate form, IAA-Aspartic acid (IAA-Asp) (D); and the expression levels of IAA biosynthetic genes,** ***TAR3*** **(B), and** ***YUC1*** **(C), in the pericarp of low and high seed weight-to-berry weight (SB) berries.** Prevéraison cluster stages were two- and one-week before véraison (2-wk PV and 1-wk PV). IAA and IAA-Asp levels were quantified by LC-MS/MS using four replicates. Gene expression was measured by qRT-PCR and expression levels are relative to low-SB berries at 2-wk PV (n = 5). Gene expressions were also measured at mid-véraison (MV) cluster stage. Significant differences between low- and high-SB at each cluster stage are indicated by asterisks (Student’s t-test, *p* < 0.05). Significant differences of each SB group between the ripening stages are denoted by different letters (lower and upper case letters are used for low- and high-SB berries, respectively (Tukey’s HSD, *p* < 0.05)).

During fruit development, auxins and cytokinins appear to be the key regulators during the maturation phase and when fruit becomes competent to ripen, ABA and ethylene play a predominant role (reviewed by [41,44]). In non-climacteric fruits that have little or no ethylene requirement for ripening, ABA has a stronger role; the decrease in auxin and the concomittant increase in ABA are significant events that signal the developmental transition to ripening [24,45,46]. To examine the emergence of auxin dynamics in low- and high-SB berries around the onset of ripening, we observed IAA levels in the pericarp of these berries separately in 2-wk PV clusters, when both were at a similar pre-ripening stage, and in 1-wk PV clusters, when ripening-related sugar accumulation had begun in low-SB berries (Figure 4). The levels of auxin in the pericarp of low-SB berries at 1-wk PV were significantly lower compared to those in high-SB berries (Figure 4A) and had already decreased to the levels of mid-véraison green berries (see Figure 2A), while those of high-SB berries remained at higher levels. High levels of auxin in developing fruit that decline before the initiation of ripening have been reported in many climacteric and non-climacteric fruits such as tomato, pepper, banana, and strawberry ([38] and reviewed by [41,47]). We observed the expression of tryptophan aminotransferase related (TAR3) and YUC flavin monooxygenase (YUC1) to determine whether the auxin levels observed follow the expression of auxin biosynthetic genes in the pericarp (Figure 4B,C). These two genes were selected based upon the literature [48] and our transcriptomic data for berry developmental stages [30], which indicates that their expression varies the most from prevéraison to early stages of véraison. The declining trend of auxin from 2-wk PV to 1-wk PV in the pericarp of low-SB berries followed the decrease in the expression of *TAR3*, but a similar decrease in expression in high-SB berries did not result in a decrease in IAA (Figure 4A and B). Unlike the expression of the *TAR* gene, the expression of the *YUC1* gene, the rate-limiting enzyme in the IPA pathway of IAA biosynthesis [49], did not differ between low- and high-SB; and its expression level in both groups was unchanged before mid-véraison (Figure 4C). Similarly, *YUC2* also showed no difference in expression between berry groups (Additional file 4). This suggests that local biosynthesis of auxin in the pericarp might be tapering off in both of the berry classes as they neared the ripening transition phase, more so in low-SB berries, and that the pericarp-synthesized auxin levels might not be very different.

An important mechanism in auxin homeostasis is its conjugation with amino acids and sugars, and in grape berries, regulation of auxin levels through conjugation with aspartic acid (IAA-Asp) by IAA-amido synthetases (GH3) has been reported ([50] and reviewed by [51]). At 2-wk PV, the levels of IAA-asp were similar in the pericarp of both low- and high-SB berries but IAA-Asp increased fourfold in low-SB berries at 1-wk PV, concomitant with the decrease in free auxin and the increase in sugar (Figures 4D). In several plants including grape, the reduction of auxin is accompanied by an increase in IAA-Asp before the onset of ripening, so IAA-Asp has been suggested as a ripening initiator, much like ABA ([52], reviewed by [47,53]). In the pericarp of high-SB berries, which did not enter into active sugar accumulation phase until mid-véraison (see Figure 3B), IAA-Asp levels remained low and unchanged across 2- and 1-wk PV. The peak expression levels of *GH3-1* and −*2* genes was at 1-wk and 2-wk PV, respectively, and decrease by mid-véraison in both groups (Additional file 4). The increase in *GH3-1* expression in low-SB berries from 2-wk to 1-wk PV matched the decrease in the levels of IAA and the increase in IAA-Asp (Figure 4A,D). However, in high-SB berries, increased *GH3-1* expression was not followed by associated changes in IAA and IAA-Asp levels (Figure 4A,D). Significant differences in the expression of *GH3* genes between low- and high-SB groups was observed at 1-wk PV, but its impact on the relative IAA and IAA-Asp levels in the pericarp of the two berry groups could not be ascertained, as our hormone analysis did not extend to mid-véraison stage. Despite the local biosynthesis and homeostasis dynamics of auxin in the pericarp, the additional hypothesized seed-sourced auxin could add substantially to the pericarp of high-SB berries, as their seed content per berry was more than twice that of low-SB berries (see Figure 3). This could potentially confound the actual local auxin levels during the ripening transition stage, as evidenced by the discrepancies observed between the expression of biosynthetic genes and actual hormone levels.

### Screening of auxin responsive genes in cultured grape cells and their induction in the pericarp of low- and high-SB berries

**Figure 5:**
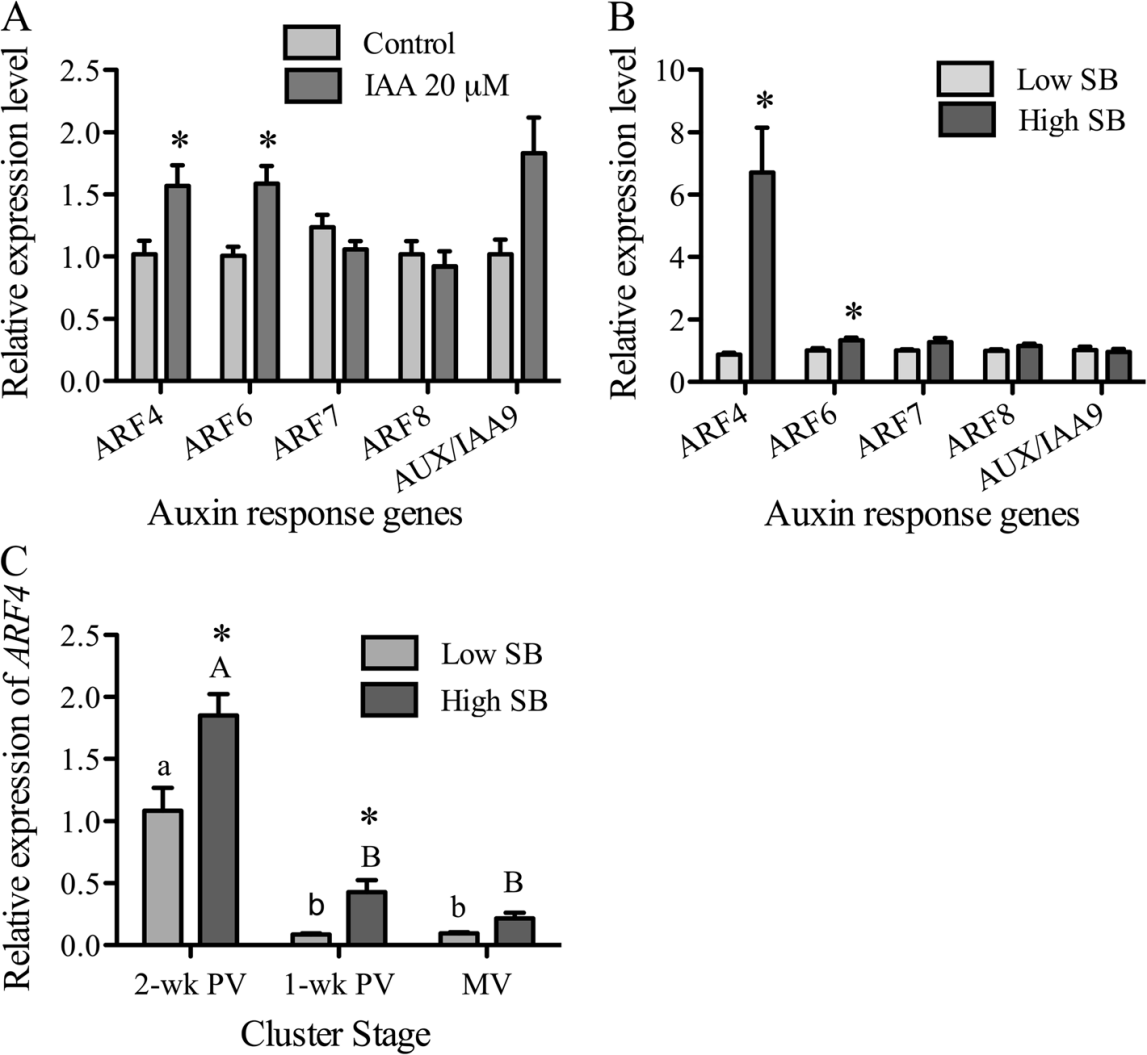
**Screening for Auxin response genes induced in grape cultured cells and in the pericarp of low and high seed containing berries. (A)** Induction of auxin response genes in cultured grape cells with auxin treatment (20 μM indole-3-acetic acid). The expression level is relative to the control. **(B)** Expression of auxin response genes in the pericarp of berries with low and high seed weight-to-berry weight (SB) from one-week prevéraison clusters. Expression level of high-SB is shown relative to that of low-SB. **(C)** Expression of *ARF4* gene in the pericarp of low and high-SB berry groups at two- and one-week prevéraison (PV), and at mid-véraison (MV). Expression levels of *ARF4* are relative to those of low-SB berries at 2-wk PV. Gene expression was analyzed by qRT-PCR and all data represent means of five replicates and error bars indicate ± SEM. Significant differences between low- and high-SB berries at each cluster stage are indicated by asterisks (Student’s t-test, *p* < 0.05). Significant differences of each SB group between the ripening stages are denoted by different letters (lower and upper case letters are used for low- and high-SB berries, respectively (Tukey’s HSD, *p* < 0.05)).

Auxin signaling regulates cell responses to different levels of auxin. The main components of signaling are auxin response factors (ARFs) that activate or repress the expression of auxin-dependent genes [54,55]. Recent evidence in other models implicates specific ARF proteins that mediate auxin responses at different stages of fruit development. Genetic studies in tomato and *Arabidopsis* have shown that ARF6, ARF7, ARF8, and AUX/IAA9 [56-59] function at the fruit initiation stage, while ARF4 is important at the ripening transition stage [60]. To examine if any of these auxin-responsive genes function in mediating the observed auxin level changes during the ripening transition in grape, expression of these *ARFs* was examined in the pericarp of low- and high-SB berries from 1-wk PV clusters, which had lower and higher auxin levels, respectively (Figure 5B). Further, we assessed the auxin-responsiveness of these genes in grape cultured cells and found that *ARF4* and *ARF6* genes were significantly induced in cells treated with 20 μM IAA for 2 h (Figure 5A). While both *ARF4* and *ARF6* had higher expression in the pericarp of high-SB berries, the expression of *ARF4* was more than sixfold higher. In *Arabidopsis* the expression of *ARF4* and *ARF19* is induced by auxin [61], and the expression of *ARF* genes that mediate auxin responses is cell- and development-context specific [62] (reviewed by [63,64]). The expression of *ARF7*, a negative regulator of auxin response that inhibits fruit set in tomato, is highest in the ovary and decreases with increasing auxin levels in this tissue [57]. Similarly in tomato, *ARF4* is preferentially expressed in fruit around the breaker stage and its expression levels follow the ripening-related auxin changes in the tissue [60]. Under-expressing *ARF4*, formerly designated *DR12*, in tomato results in dark green, immature fruits and up-regulates the expression of sugar metabolism-related genes [60,65]. Based upon this evidence, our results indicate that *ARF4* mediates the response to auxin changes during the grape berry ripening initiation, and is likely a negative regulator of the ripening-related changes in the pericarp during véraison.

Expression trends for *ARF4* in low- and high-SB berries show the greatest difference between both berry groups at 1-wk PV (Figure 5C), when the low-SB berries begin rapid accumulation of sugars (see Figure 3B). However, differences in *ARF4* expression between the berry groups was obvious at 2-wk PV, when the sugars were at very similar levels and both groups were presumably at the same pre-ripening stage. The down-regulation of *ARF4* in the pericarp of low-SB berries from 2- to 1-wk PV was more than twelve fold (Figure 5C), concomitant with the steepest drop in the IAA levels and the increase of sugar (Figures 4 and 3). In high-SB berries the expression level of *ARF4* reached its lowest at mid-véraison (Figures 5C). Overall, the expression of *ARF4* follows the same declining trend as auxin in the pericarp, reaching its minimum level towards véraison from its maximum at 2-wk PV, and coinciding with the increased expression of ripening-promoting genes discussed in the following section. These results indicate that the role of *ARF4* during the grape berry ripening transition is similar to its reported role in tomato. Its expression is also indicative of the changes in the pericarp auxin levels of high and low-SB berries. Auxin transporters are known to be involved in the transport of auxin from seed to pericarp and a spatial distribution gradient of auxin flux has been shown [15] (reviewed by [66]). In non-climacteric strawberry, removal of achenes enhances ripening and this enhancement could be abolished by application of auxin [16]. These observations might suggest that auxin from seeds contributes to the differential regulation of ripening-related signaling in the pericarp of grape berries.

### Levels of ABA and ABA-related transcripts in low and high-SB berries

**Figure 6:**
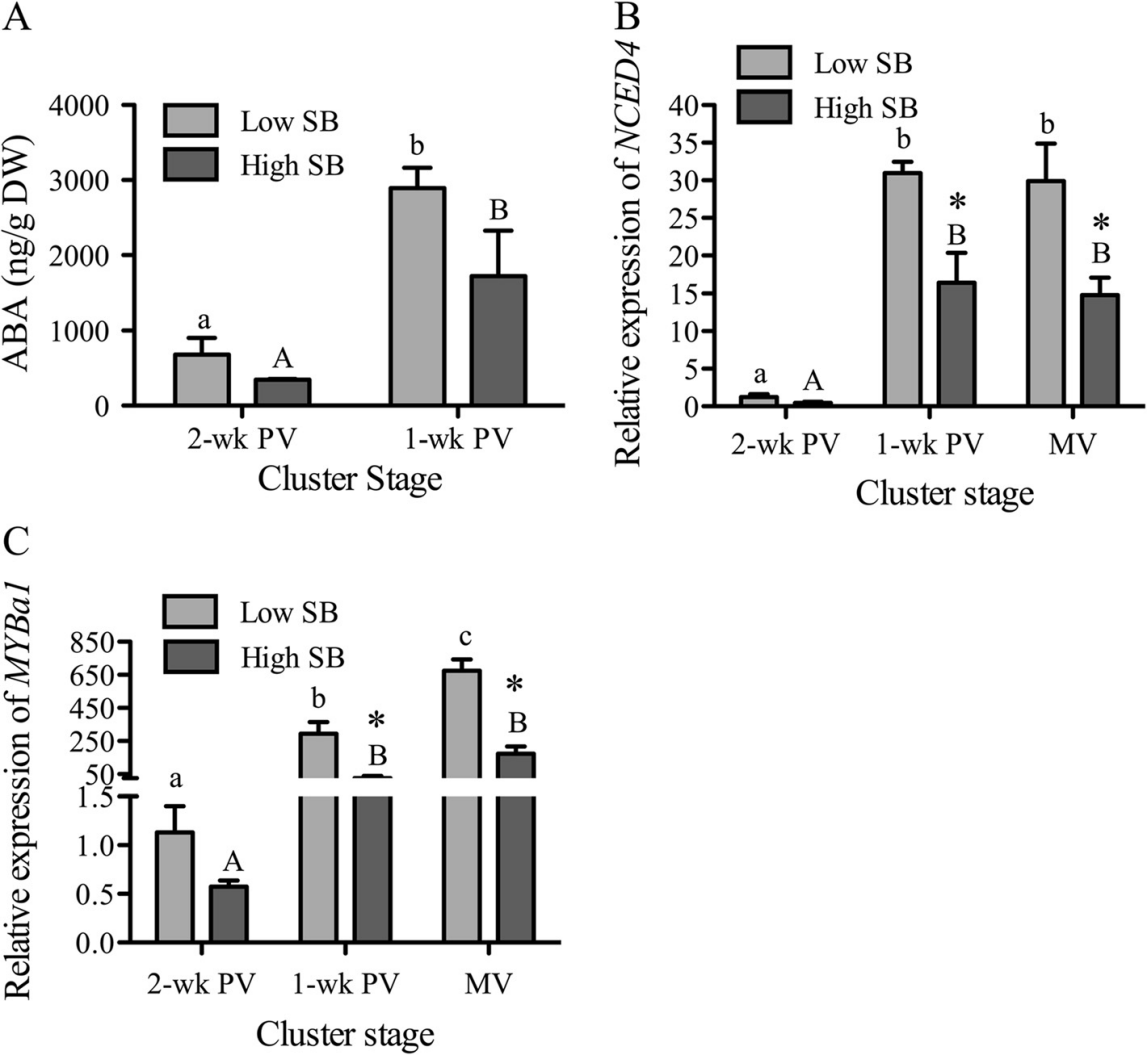
**Hormone level of abscisic acid (ABA) (A), and the expression levels of ABA biosynthesis gene,** ***NCED4*** **(B) and ABA-responsive gene,** ***MYBa1*** **(C) in the pericarp of low and high seed weight-to-berry weight (SB) groups.** ABA levels were quantified by LC-MS/MS using four replicates. Gene expressions were measured by qRT-PCR and expression levels are relative to those of low-SB berries at 2-wk PV (n = 5). Error bars indicate ± SEM. Prevéraison stages were two- and one-week before véraison (2-wk PV and 1-wk PV). Gene expressions were also measured at mid-véraison (MV) cluster stage. Significant differences between low- and high-SB berries at each cluster stage are indicated by asterisks (Student’s t-test, *p* < 0.05). Significant differences of each SB group between the ripening stages are denoted by different letters (lower and upper case letters are used for low- and high-SB berries, respectively (Tukey’s HSD, *p* < 0.05)).

The consequences of changes in auxin levels in the pericarp vary depending on the stage of the fruit development. During early fruit development, seed-sourced auxin acts as a signal to initiate gibberellin (GA) biosynthesis and signaling in the pericarp (reviewed by [9]), where *ARF7* expression mediates auxin-GA crosstalk [67]. In the pericarp of pea, auxin application after removal of seeds restores GA biosynthesis and pericarp growth, which supports the hypothesis that auxin is transported from the seeds [68]. At around ripening initiation stage, auxin represses genes involved in ripening, such as cell-wall modifying proteins, and those involved in sugar metabolism, and anthocyanin biosynthesis in the pericarp [20,60,69,70]. In grape, ABA is the hormone involved in the initiation of ripening-related changes, as ethylene is in climacteric fruits. Changes in ABA levels and the expression of the main ABA biosynthetic gene, 9-*cis*-epoxycarotenoid dioxygenase (*NCED*), and that of an ABA-responsive *MYBA1*, a transcription factor involved in the anthocyanin biosynthesis, were examined in the pericarp of low- and high-SB berries (Figure 6). Like IAA, early differences in ABA were observed between low- and high-SB berries (Figure 6A). The pericarp of low-SB berries exhibited a trend towards a higher concentration of ABA, though statistically non-significant, compared to that of high-SB berries as early as two weeks prior to mid-véraison. The early increase in ABA is probably in direct response to the earlier decrease in auxin in the pericarp of low-SB berries (Figure 4A). An inhibitory influence of auxin on ABA during ripening has been suggested in several fruit models, and the down-regulation of *NCED* expression by auxin has been reported [71,72]. The expression level of *NCED4* was significantly higher in the pericarp of low-SB berries compared to that in high-SB by 1-wk PV, suggesting a higher level of ABA synthesis (Figure 6B). These results indicate that, depending on relative seed content, the differences in ripening-related hormones emerge in the pericarp much earlier than the onset of ripening, which predispose the berries with lower seed content to ripen earlier compared to those with higher seed content.

Similar to *ARF4*, differences in the expression of the ABA-responsive *MYBa1* gene between low- and high-SB berries were apparent from 2-wk PV and became more pronounced at 1-wk PV. The expression of *MYBa1* in the pericarp of high-SB berries was significantly lower and remained comparatively lower until mid-véraison. At this stage, auxin levels had decreased and ripening initiation had started in low-SB berries while auxin levels were still higher in relatively immature high-SB berries (see Figure 4A). In strawberry, a non-climacteric fruit like grape, expression of *NCED2* and *MYB10* are repressed by auxin and activated by ABA [70,72]. Further, removal of achenes, the source of auxin, increases *MYB* expression, which can be abolished by auxin application [70]. Similarly, tomato transgenic lines under-expressing *ARF4* show enhanced expression of *golden2-like* (*SlGLK*), a MYB-type transcription factor [60]. To check whether these genes are direct targets of ARF4, we performed the *in silico* analysis of the promoters for auxRE binding sites. Analysis of the 2-KB promoter sequence of *MYBa1* identified only the expected ABA-response elements. However, *NCED4* possesses a conserved auxRE, TGTCTC, at position −803 and an auxin induction element, ACTTTA, at position −1351 (data not shown), suggesting possible negative regulation of its expression by auxin. In the 2-wk PV cluster, both low- and high-SB berries were at a similar immature pre-ripening physiological stage with no sugar accumulation and only differed in their seed content (Figure 3). Our results suggest a relationship between low seed content and the resulting earlier decreased auxin levels in the pericarp of low-SB berries and the observed higher expression of ABA-related genes that leads to their earlier ripening.

### Exogenous IAA and ABA treatment of prevéraison clusters and changes in the composition of green, pink, and red berries in low- and high-SB groups

**Figure 7:**
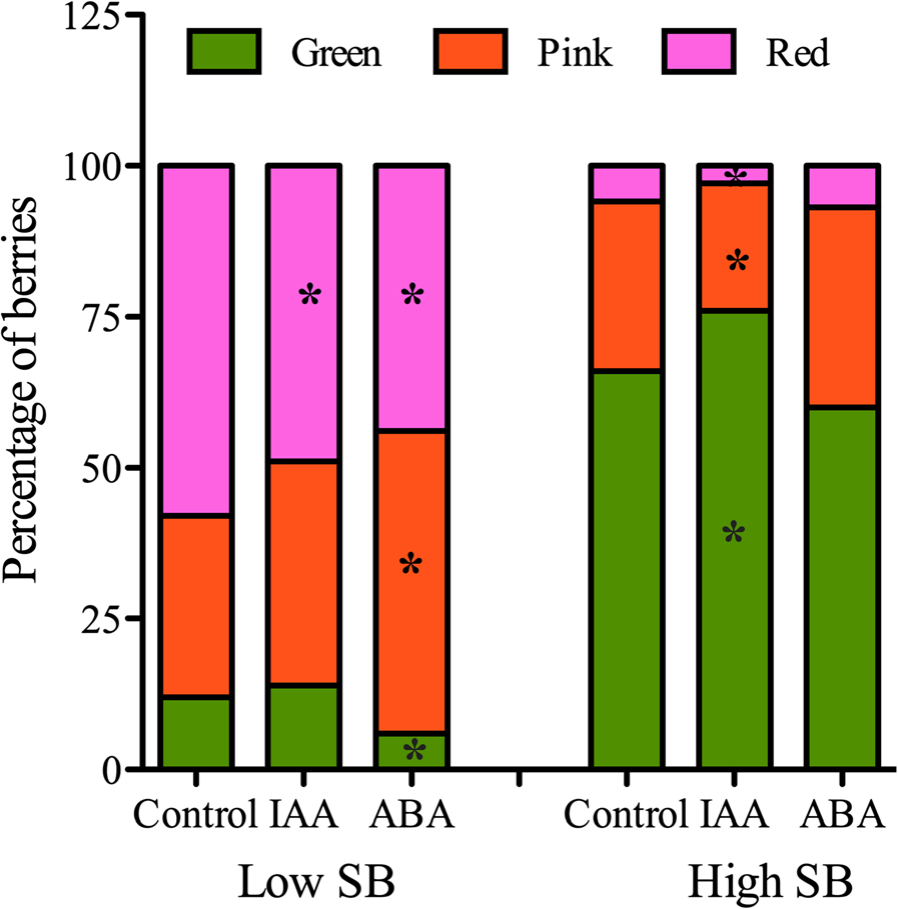
**Effect of indole-3-acetic acid (IAA) and abscisic acid (ABA) treatments on the composition of ripening classes in low and high seed-to-berry weight (SB) groups.** Eight days before the expected mid-véraison, two clusters each on three different plants were treated with 50 mg/L hormone solutions in Tween-20. Control plants were sprayed with 0.01% Tween-20 alone. SB ratio was calculated for each berry of the clusters at mid-véraison and the numbers of green, pink, and red berries in low- and high-SB groups were counted (about 300 berries per treatment). Approximate test for equal proportions was used to identify differences in the distribution of the ripening classes between control and treated and significant differences were marked with asterisks (*p* < 0.05).

To evaluate the influence of seed content on the timing of ripening initiation through hormone signals, exogenous applications of hormones were performed on clusters one week before expected véraison. The normal proportions of berries transitioning into the progressive véraison-ripening stages of green, pink, and red, which follow the SB ratio, should be perturbed when the levels of ripening-related hormones in the pericarp are altered. The emergence of normal proportions of green, pink, and red berries in IAA-treated clusters at mid-véraison was not different from that of control, while in ABA-treated clusters, fewer berries stayed green and more transitioned to pink and red stages (Additional file 5). However, when the proportions of the three ripening stages in low- and high-SB berry groups were examined separately, for which berries with low and high seed content should yield mostly colored and green berries, respectively (Figure 1B), significant changes were observed (Figure 7). ABA treatment disrupted the proportions of ripening stages most significantly in low-SB group, while IAA mostly affected the high-SB berry group (Figure 7). Decreases in the pericarp IAA level and increased synthesis of ABA are required for the onset of ripening in many non-climacteric fruits [24,38]. One week before véraison, at the time of hormone treatment, IAA had not reached yet its basal level in high-SB berries and berries were probably not in the irreversible phase of the ripening program. Exogenous IAA likely further elevates the pericarp IAA levels and results in more berries staying green and pink that otherwise would have advanced in ripening, as shown by other studies [20,21,72]. Ripening-related expression of genes associated with ABA and pigment biosynthesis, and with cell wall loosening is generally inhibited by higher auxin levels [19]. On the other hand, berries with low seed content, owing to the earlier decrease in IAA in the pericarp, had already entered ripening initiation at the time of IAA treatment, which made them less responsive to exogenous IAA application. But fewer berries transitioning from pink to red stage compared to control clusters were observed. ABA treatment caused enhanced green to pink and red stage transitions in low-SB berries and the treated clusters showed significantly lower number of green low-SB berries. On the other hand, no changes in the proportions of berries of each color in the high-SB group could be due to higher auxin levels. These results show that the timing of the ripening transition, which depends on both the auxin decrease and the ABA increase in the pericarp, is influenced by the relative seed content and its possible auxin contribution to the pericarp, and that this mechanism can be uncoupled by external sources of auxins.

## Conclusions

The influence of seeds on pericarp ripening through the transport of growth-regulating hormones has been demonstrated in tomato and strawberry. In grape, the influence of seed on berry growth has been extensively studied, but its influence on the ripening has not been elucidated. We investigated the role of seeds in the asynchronous nature of ripening among the berries of a grape cluster at the onset of ripening. Berries with higher seed content enter the ripening phase 4 to 14 d later than berries with lower seed content. In addition, the linear correlation between the seed weight-to-berry weight ratio (SB) and berry sugar levels implicates the seed in the regulation of the ripening transition. During this stage, 86% of berries with low SB are in the rapid sugar-accumulation stage and change color, whereas 78% of berries with high-SB remain green with basal sugar levels. Differences in auxin and ABA levels in the pericarp of low- and high-SB berry groups begin to emerge towards the end of the fruit maturation phase, at least two weeks pre-ripening, whereas auxin decreases and ABA increases are delayed in high-SB berries. Higher expression of auxin-inducible *ARF4* in the pericarp of high-SB berries compared to that in low-SB berries at ripening initiation suggests that *ARF4* might play a role in mediating the ripening-related auxin responses in grape berry. Overall, the quantitative relationship between berry seed content and pericarp auxin levels, high auxin levels in seeds, the delaying effect of higher seed content on ripening initiation, and the transport of auxin from seed to pericarp reported in other fruit models together suggest that relative seed content is a major factor in the timing of entry of grape berries into the ripening phase, and provide an explanation for the asynchronous ripening nature of a grape cluster.

## Methods

### Plant materials and sampling

Experiments were conducted during 2012–2013 at the Oregon State University research experimental station (Monroe, OR). *Vitis vinifera* L. cv. Pinot noir clone ‘Pommard’ grafted to 101–14 rootstock, trained in a double guyot system with vertically positioned shoots, was used for these experiments. Primary clusters from five vines were used for these experiments. Clusters were sampled at two stages of prevéraison, including late lag phase and second growth phase, when all of the berries were green, and at mid-véraison, when 50% of the berries in the cluster had changed color. Mid-véraison stage was reached at approximately 69 days post-anthesis for all clusters. Clusters sampled at each stage were transported to the laboratory on dry ice and stored at −80°C until further analysis and were always maintained on dry ice during the workflow of the analysis. Each berry was weighed and dissected to separate the seed and pericarp tissues in a brief semi-thawed state and the seeds from each berry were weighed. A longitudinal section of frozen pulp tissue was used to measure the sugar level (Total soluble solids, °Brix) of the berry using a digital refractometer (SPER Scientific Inc., USA) before storing all the tissues at −80°C. Berry sampling and analysis methods followed for the assessment of ripening delay times of underripe berries in Additional file 1 have been described elsewhere [30].

### Data analysis and selection of low and high seed weight-to-berry weight ratio (SB) berries

Grape clusters from prevéraison to mid-véraison stages were collected to observe biochemical and growth changes, and differences in hormone levels and gene expression between berries of low- and high-SB groups. A minimum of three to four clusters from each stage were used in the study and all the berries of the clusters were sampled to measure berry weight, seed weight, seed number, and sugar level of individual berries. The seed weight-to-berry weight ratio (SB) was calculated for individual berries and pericarp weight was derived by subtracting the seed weight from the whole berry weight. From the range of SB values for each cluster stage, 40% each of the berries in the higher and lower ranges were classified as high- and low-SB, respectively. Final numbers of berries in each of the high- and low-SB categories were about 150 berries at all three stages.

### Induction of auxin-response genes in cell culture experiments

Cell suspension cultures of *Vitis vinifera* (L.) cv. Gamay Fréaux var. ‘Teinturier’ were maintained in the maintenance medium as previously described [73]. For auxin treatment experiments, 7-day-old cell cultures were inoculated into a fresh medium at 1:4 (v:v) and allowed to grow for 3 d. Four replicate cultures were treated with indole-3-acetic acid solution (Sigma Life Sciences) solution in methanol (w/v), which was diluted in 1 mL of maintenance medium to achieve a final IAA concentration of 20 μM and control cultures received an equal volume of methanol in maintenance medium. Control and IAA-treated cells were harvested after 2 h by filtration under vacuum, rapidly washed with fresh medium, flash frozen in liquid nitrogen and stored at −80°C. Total RNAs extracted from the frozen cells were used to study the expression of IAA-induced genes.

### Extractable seed tannin assay

Tannin levels in seeds were measured using a methyl cellulose-precipitable tannin assay [74]. Seeds from five berries representative of low- and high-SB groups that exhibited maximum differences in seed weight-to-berry weight ratio were selected for the assay. Seeds from each biological replicate were homogenized in liquid nitrogen and approximately 100 mg of fresh tissue were extracted for 1–2 h with 1 mL of 50% ethanol. Two technical replicates for each extracted sample were used. The appropriate volume of the extract to use in 1 mL reaction was determined through a series of dilutions. The reaction contained the seed extract, 0.04% methyl cellulose solution, and a saturated solution of ammonium sulfate, while parallel blank reactions contained no methyl cellulose polymer. Tannins were precipitated by centrifugation at 14,000 rpm for 10 min and the absorbances of the supernatant for blank and methyl cellulose-containing reactions were measured at 280 nm in glass cuvettes using a Genesis 10S UV–vis spectrophotometer (Thermo Scientific, USA). Epicatechin (Sigma-Aldrich, St. Louis, USA) solutions at different concentrations were used to establish a calibration curve for reporting tannin concentrations as epicatechin equivalents.

### Hormone analysis in seed and pericarp tissues

For hormone analysis in the pericarp tissue of high- and low-SB berries, five individual berries each from low- and high-SB groups that had high differences in SB were used as biological replicates. Pericarp tissues of the selected berries were used for the quantification of abscisic acid and auxin analytes (IAA and IAA-Aspartic acid) using LC-MS/MS under multiple-reaction monitoring mode following the established method for grape berries [75]. For hormone levels in skin, pulp and seed tissues, presented in Figure 2, the experiment was conducted in 2010–2011. Pre-véraison berries were collected 54 d after anthesis and mid-véraison-green, −pink, and -red berries were collected 69 d post anthesis. Five clusters each on four different plants were used for sampling and five berries belonging to each ripening class were sampled from each of five clusters from each plant to make a replicate. Skin, pulp, and seed were separated while the berries were still frozen. Homogenized and freeze-dried tissues including deuterated internal standards for each analyte were extracted in methanol:formic acid:water (15:1:4, v:v:v) at 4°C for 20 h. The extract was cleaned using the solid phase extraction properties of Oasis HLB SPE and Oasis MCX cartridges (Waters, Mildford, MA, USA) (Waters, USA). ABA and auxin analytes bound to MCX were eluted with 100% methanol and the eluate was evaporated overnight and reconstituted with acetonitrile:water:formic acid (15:85:0.1, v:v:v) for analysis. Chromatography separation was carried out using an Agilent Zorbax Extend-C_18_ column (2.1 mm × 150 mm; 5 μm) using a binary gradient of acetonitrile, water, and 0.1% formic acid with a gradient program of 40 min duration. Acquisition of the mass spectral data was performed on a hybrid triple quadrupole/linear ion trap 4000 QTrap LC-MS/MS instrument equipped with a Turbo V source (Applied Biosystems ®, Life Technologies, NY, USA). Mass spectra for ABA were acquired in the negative mode while the mass spectra for auxin compounds were acquired in the positive mode, and the analysis was performed using Analyst software version 1.5.1 (Applied Biosystems, USA). Analyte concentrations were calculated against calibration curves and expressed as nanograms per gram dry weight of the tissue.

### Hormone spray experiment in the prevéraison clusters

Eight days before mid-véraison, two selected primary clusters each from three plants were treated with either indole-3-acetic acid (Sigma) (50 mg/L), (+)- *cis, trans*-abscisic acid (A.G. Scientific Inc., CA, USA) (50 mg/L) solutions in 0.01% Tween-20®, or Tween-20 alone. The clusters were sampled at mid-véraison and the same workflow explained above was followed to record the ripening phenotype using visual color observation, berry weight, and seed weight for each berry.

### RNA isolation and cDNA synthesis

The same individual berries used for the hormone analyses were processed for gene expression. Total RNAs from pericarp tissue were isolated using the RNeasy Mini Kit (Qiagen Inc., Valencia, CA, USA). Because of the high sugar and phenolic content of the tissues, Qiagen RLC buffer (2% polyethylene glycol (MW 20,000), 0.2 M sodium acetate (pH 5.2), and 1% β-mercaptoethanol was substituted for lysis buffer. For the remainder of the procedure, the manufacturer’s protocol was followed, including on-column DNase digestion (RNase-free DNase, Qiagen, Valencia, USA). The quality and integrity of RNA prepared RNA was assessed using 280/260 and 230/260 ratios and on agarose gel. First-strand cDNA was synthesized from 1 μg total RNA using SuperScript III Reverse Transcriptase (Invitrogen, Carlsbad, USA) and oligo (dT)_12–18_ primers in a 20-μl reaction volume. Five microliters of the 5x- diluted cDNA was used as template in RT-PCR reactions.

### Quantitative real-time RT-PCR analysis

Gene expression levels were analyzed with the QuantiFast SYBR Green (Qiagen) assay using an ABI 7500 Fast Real-Time PCR System (Applied Biosystems). The cDNAs prepared from the five individual berries were used as biological replicates for each SB group and each PCR reaction was performed in duplicate. The peptidyl-prolyl *cis-trans* isomerase gene (VIT_06s0004g06610), based on its low M value [76] in pulp tissues between prevéraison and mid-véraison stages was used for data normalization. Oligonucleotide gene-specific primer pairs were designed with Primer 3 software (http://biotools.umassmed.edu/bioapps/primer3_www.cgi) so that the forward and reverse primers are located in the coding region and 3′ untranslated region respectively (Additional file 6). The size of the amplicons was generally between 100-125 bp. The reaction conditions were: heat activate/denature at 95°C for 5 min (one cycle); followed by 95°C for 10 s, 60°C for 30 s (40 cycles). The specificity of the primers was assessed by PCR on agarose gel and that of the real-time PCR reactions was confirmed by melting curve analysis. The amplification products were verified by sequencing (Center for Genome Research and Biocomputing, OSU). Data were acquired and exported with 7500 Fast Software version 2.0.6 (Applied Biosystems) and relative gene expression was calculated using the ΔΔC_t_ method. Relative fold-expression for each gene was calculated relative to the level of expression in low-SB berries at 2-wk PV.

### Statistical analysis

Student’s t-test was applied to the data for comparisons between low- and high-SB berries (P < 0.05). A one-way ANOVA was conducted to compare the differences in parameters in each berry group between cluster stages followed by post-hoc means comparison using Tukey’s HSD test (P < 0.05). For hormone spray experiments, an approximate test for equal proportions [77] was used to identify significant differences in the distribution of berry ripening classes (green, pink, or red) among low- and high-SB berry groups in treated clusters compared to those of control.

## Additional files

Additional file 1:
**Ripening lag in green hard, green soft, pink compared to red berries around véraison.** Progression in the accumulation of sugars and pigments in pink, green soft, and green hard berries of the mid-véraison cluster were followed to post-mid-véraison stage and the times the under-ripe berries reach sugar and color levels in red berries at mid-véraison (indicated by boxed text) were calculated [30]. The horizontal pink, light green, and dark green bars at the bottom of the plot indicate the duration of time taken by pink, green soft, and green hard berries, respectively to reach the sugar and color equivalent levels of mid-véraison-red berries. Methods followed to calculate these times and color index to measure the color level were described elsewhere [30]. Additional file 2:
**Individual seed weight in green, pink and red berries of véraison cluster.** The mean calculation per ripening class is based on more than 300 individual berries. Error bars indicate ± SEM. Different letters denote significant difference (Tukey’s HSD test, *p* < 0.05). Additional file 3:
**Changes in (A) Seed weight-to-berry weight ratio (SB), (B) Sugar levels, (C) Seed weight, and (D) pericarp weight between low and high-SB berries of the cluster from two week-prevéraison (PV) to 100% véraison (V).** Data represent means of all the berries with low- and high-SB ranges (see Methods), respectively. Number of berries is approximately a minimum of 150 for each of low and high-SB group at each cluster stage. In panel D, the ripening phenotype of the berries of low and high-SB groups were indicated as dark green (green immature phase), light green (green soft phase with sugar increase), red (pink phase), and purple (red phase). Error bars represent ± SEM. Significant differences between low- and high-SB at each cluster stage are indicated by asterisks (t-Test, *p* < 0.05). Significant differences for each SB group between the ripening stages are denoted by different letters (lower and upper case letters are used for low and high-SB berries, respectively (Tukey’s HSD, *p* < 0.05)). Additional file 4:
**Expression of (A) YUC2, (B) GH3-1, and (C) GH3-2 in the pericarp of low and high-SB berries.** Gene expression was measured by qRT-PCR and expression levels are presented relative to those of low-SB berries at 2-wk PV. All data represent means of five replicates and error bars indicate ± SEM. Prevéraison stages were two- and one-week before véraison (2-wk PV and 1-wk PV), and mid-véraison (MV). Significant differences between low and high-SB groups at each cluster stage are indicated by asterisks (t-Test, *p* < 0.05). Significant differences of each SB group between the ripening stages are denoted by different letters (lower and upper case letters are used for low and high-SB berries, respectively (Tukey’s HSD, *p* < 0.05)). Additional file 5:
**Percentages of green, pink, and red berries in hormone-treated mid-véraison clusters.** Two clusters each on three different plants were treated with 50 mg/L solutions of indole-3-acetic acid or abscisic acid in Tween-20, or 0.01% Tween-20 one week before the expected mid-véraison. The clusters were then harvested at mid-véraison and green, pink, and red berries counted (about 300 berries per treatment). Approximate test for equal proportions was used to identify differences in the distribution of the ripening classes between control and treated clusters, and significant differences were marked with asterisks (*p* < 0.05). Additional file 6:
**Vitis gene IDs and sequences of the primers used in the qRT-PCR experiment.**

## Abbreviations

2-wk PV: Two weeks before véraison
1-wk PV: One week before véraison
MV: Mid-véraison stage
SB: Seed weight-to-berry weight ratio
IAA: Indole-3-acetic acid
ABA: Abscisic acid
IAA-Asp: IAA-Aspartic Acid
TAR: Tryptophan aminotransferase related
YUC: YUCCA
GH3: Gretchen Hagen3
NCED: 9-*cis*-epoxycarotenoid dioxygenase
