# Supplementary material for: Timing of ripening initiation in grape berries and its relationship to seed content and pericarp auxin levels
Source: BMC Plant Biol. 2015 Feb 12;15:46. doi: 10.1186/s12870-015-0440-6 (PMC4340107; doi:10.1186/s12870-015-0440-6)
Supplement: Additional file 1: — Ripening lag in green hard, green soft, pink compared to red berries around véraison. Progression in the accumulation of sugars and pigments in pink, green soft, and green hard berries of the mid-véraison cluster were followed to post-mid-véraison stage and the times the under-ripe berries reach sugar and color levels in red berries at mid-véraison (indicated by boxed text) were calculated [30]. The horizontal pink, light green, and dark green bars at the bottom of the plot indicate the duration of time taken by pink, green soft, and green hard berries, respectively to reach the sugar and color equivalent levels of mid-véraison-red berries. Methods followed to calculate these times and color index to measure the color level were described elsewhere [30]. [file 12870_2015_440_MOESM1_ESM.pdf]

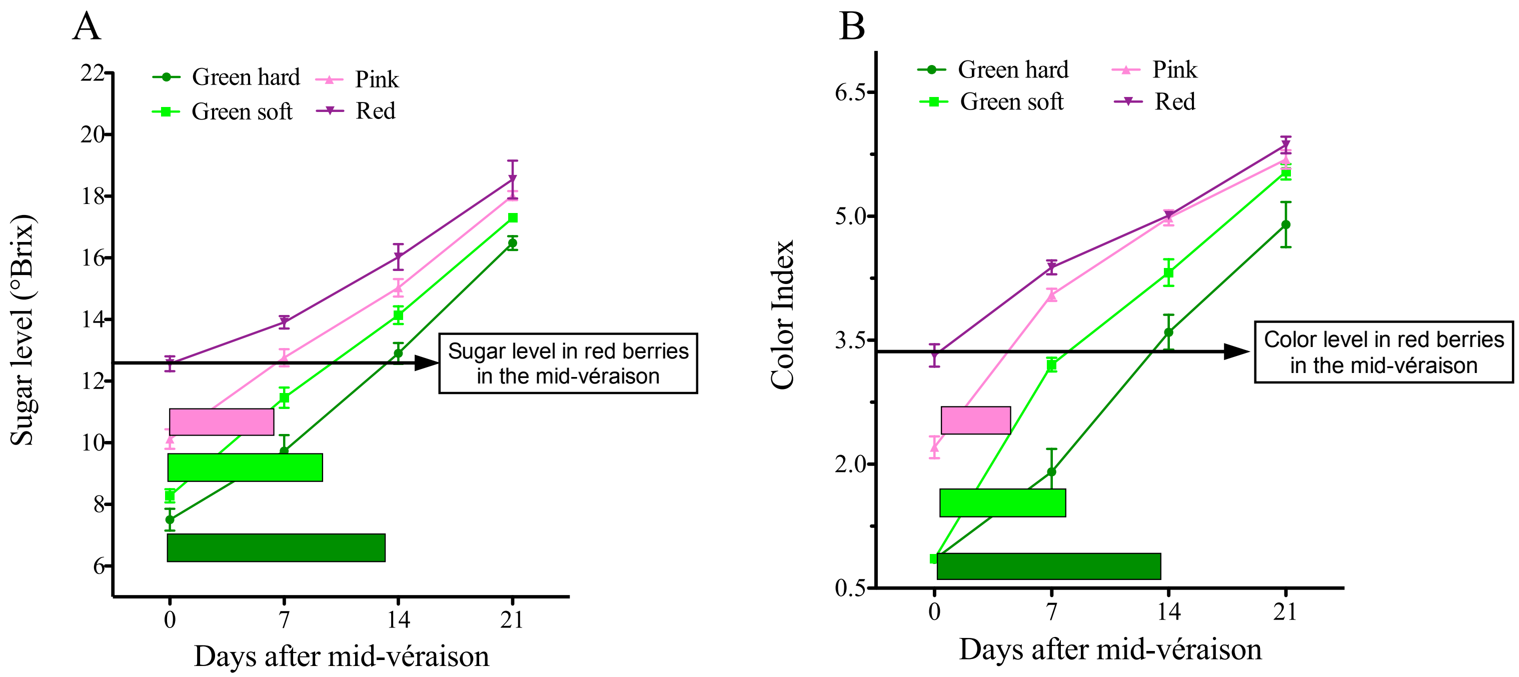

Additional file 1: Ripening lag in green hard, green soft, pink compared to red berries around véraison. Progression in the accumulation of sugars and pigments in pink, green soft, and green hard berries of the mid-véraison cluster were followed to post-mid-véraison stage and the times the under-ripe berries reach sugar and color levels in red berries at mid-véraison (indicated by boxed text) were calculated [30]. The horizontal pink, light green, and dark green bars at the bottom of the plot indicate the duration of time taken by pink, green soft, and green hard berries, respectively to reach the sugar and color equivalent levels of mid-véraison-red berries. Methods followed to calculate these times and color index to measure the color level were described elsewhere [30].
