# Supplementary material for: Timing of ripening initiation in grape berries and its relationship to seed content and pericarp auxin levels
Source: BMC Plant Biol. 2015 Feb 12;15:46. doi: 10.1186/s12870-015-0440-6 (PMC4340107; doi:10.1186/s12870-015-0440-6)
Supplement: Additional file 2: — Individual seed weight in green, pink and red berries of véraison cluster. The mean calculation per ripening class is based on more than 300 individual berries. Error bars indicate ± SEM. Different letters denote significant difference (Tukey’s HSD test, p < 0.05). [file 12870_2015_440_MOESM2_ESM.pdf]

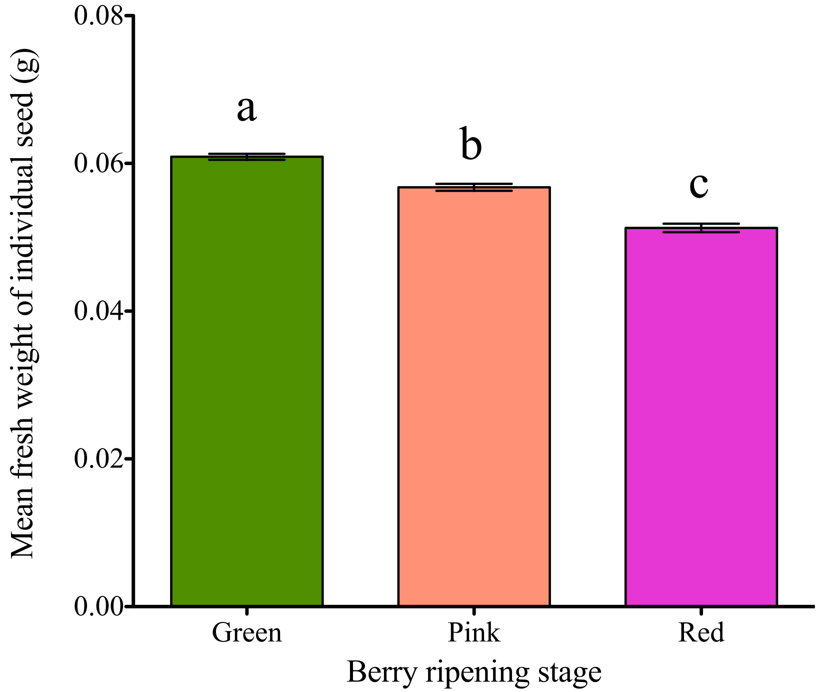

Additional file 2: Individual seed weight in green, pink and red berries of véraison cluster. The mean calculation per ripening class is based on more than 300 individual berries. Error bars indicate ± SEM. Different letters denote significant difference (Tukey's HSD test, $p < 0.05$).
