# Supplementary material for: Timing of ripening initiation in grape berries and its relationship to seed content and pericarp auxin levels
Source: BMC Plant Biol. 2015 Feb 12;15:46. doi: 10.1186/s12870-015-0440-6 (PMC4340107; doi:10.1186/s12870-015-0440-6)
Supplement: Additional file 3: — Changes in (A) Seed weight-to-berry weight ratio (SB), (B) Sugar levels, (C) Seed weight, and (D) pericarp weight between low and high-SB berries of the cluster from two week-prevéraison (PV) to 100% véraison (V). Data represent means of all the berries with low- and high-SB ranges (see Methods), respectively. Number of berries is approximately a minimum of 150 for each of low and high-SB group at each cluster stage. In panel D, the ripening phenotype of the berries of low and high-SB groups were indicated as dark green (green immature phase), light green (green soft phase with sugar increase), red (pink phase), and purple (red phase). Error bars represent ± SEM. Significant differences between low- and high-SB at each cluster stage are indicated by asterisks (t-Test, p < 0.05). Significant differences for each SB group between the ripening stages are denoted by different letters (lower and upper case letters are used for low and high-SB berries, respectively (Tukey’s HSD, p < 0.05)). [file 12870_2015_440_MOESM3_ESM.pdf]

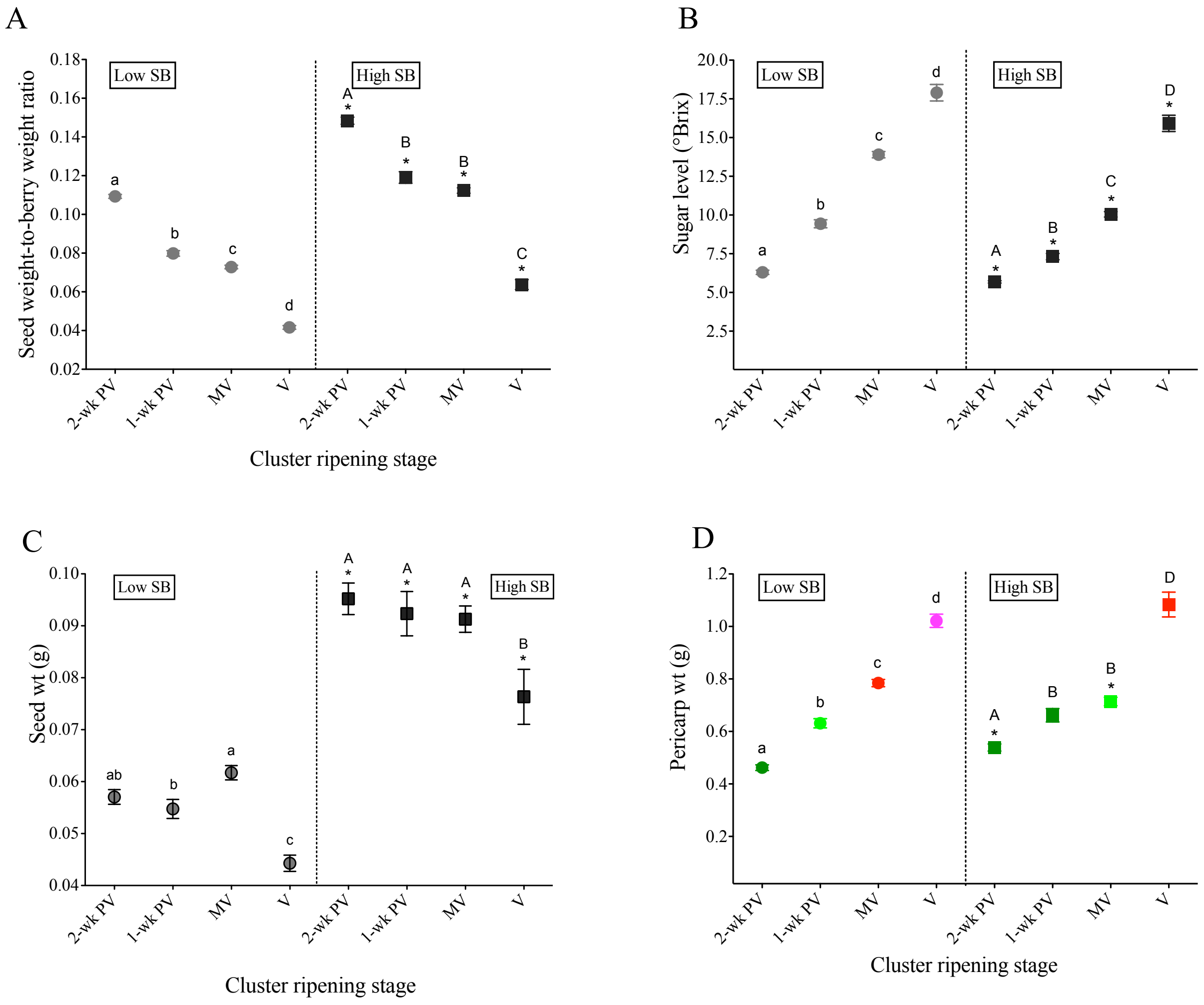

Additional file 3: Changes in (A) Seed weight-to-berry weight ratio (SB), (B) Sugar levels, (C) Seed weight, and (D) pericarp weight between low- and high-SB berries of the cluster from two week-prevéraison (PV) to 100% véraison (V). Data represent means of all the berries with low- and high-SB ranges (see Methods), respectively. Number of berries is approximately a minimum of 150 for each of low and high-SB group at each cluster stage. In panel D, the ripening phenotype of the berries of low and high-SB groups were indicated as dark green (green hard immature phase), light green (green soft phase with sugar increase), red (pink phase), and purple (red phase). Error bars represent ±SEM. Significant differences between low- and high-SB at each cluster stage are indicated by asterisks (t-Test, $p < 0.05$). Significant differences for each SB group between the ripening stages are denoted by different letters (lower and upper case letters are used for low- and high-SB berries, respectively (Tukey's HSD, $p < 0.05$)).
