# Supplementary material for: Timing of ripening initiation in grape berries and its relationship to seed content and pericarp auxin levels
Source: BMC Plant Biol. 2015 Feb 12;15:46. doi: 10.1186/s12870-015-0440-6 (PMC4340107; doi:10.1186/s12870-015-0440-6)
Supplement: Additional file 4: — Expression of (A) YUC2, (B) GH3-1, and (C) GH3-2 in the pericarp of low and high-SB berries. Gene expression was measured by qRT-PCR and expression levels are presented relative to those of low-SB berries at 2-wk PV. All data represent means of five replicates and error bars indicate ± SEM. Prevéraison stages were two- and one-week before véraison (2-wk PV and 1-wk PV), and mid-véraison (MV). Significant differences between low and high-SB groups at each cluster stage are indicated by asterisks (t-Test, p < 0.05). Significant differences of each SB group between the ripening stages are denoted by different letters (lower and upper case letters are used for low and high-SB berries, respectively (Tukey’s HSD, p < 0.05)). [file 12870_2015_440_MOESM4_ESM.pdf]

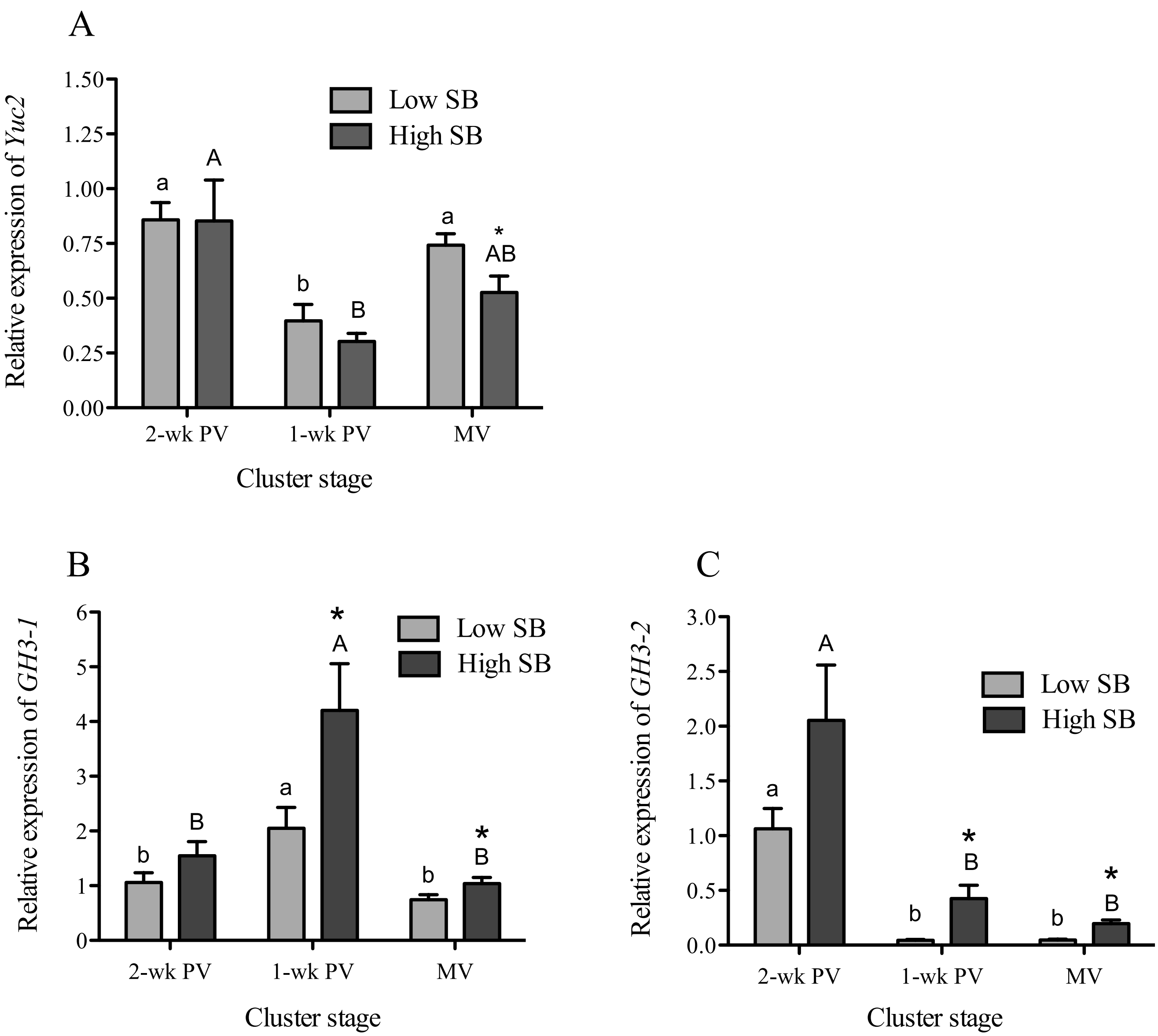

Additional file 4: Expression of (A) *YUC2*, (B) *GH3-1*, and (C) *GH3-2* in the pericarp of low- and high-SB berries. Gene expression was measured by qRT-PCR and expression levels are presented relative to those of low-SB berries at 2-wk PV. All data represent means of five replicates and error bars indicate ± SEM. Pre-véraison stages were two- and one-week before véraison (2-wk PV and 1-wk PV), and mid-véraison (MV). Significant differences between low and high SB groups at each cluster stage are indicated by asterisks (t-Test, $p < 0.05$). Significant differences of each SB group between the ripening stages are denoted by different letters (lower and upper case letters are used for low- and high-SB berries, respectively (Tukey's HSD, $p < 0.05$)).
