# Supplementary material for: Timing of ripening initiation in grape berries and its relationship to seed content and pericarp auxin levels
Source: BMC Plant Biol. 2015 Feb 12;15:46. doi: 10.1186/s12870-015-0440-6 (PMC4340107; doi:10.1186/s12870-015-0440-6)
Supplement: Additional file 5: — Percentages of green, pink, and red berries in hormone-treated mid-véraison clusters. Two clusters each on three different plants were treated with 50 mg/L solutions of indole-3-acetic acid or abscisic acid in Tween-20, or 0.01% Tween-20 one week before the expected mid-véraison. The clusters were then harvested at mid-véraison and green, pink, and red berries counted (about 300 berries per treatment). Approximate test for equal proportions was used to identify differences in the distribution of the ripening classes between control and treated clusters, and significant differences were marked with asterisks (p < 0.05). [file 12870_2015_440_MOESM5_ESM.pdf]

Additional file 5: Percentages of green, pink, and red berries in hormone-treated mid-véraison clusters.

Treatment	Green	Pink	Red
Control	40.8	29.3	29.8
IAA	42.1	29.7	28.2
ABA	34.2*	40.8*	25.1

Two clusters each on three different plants were treated with 50 mg/L solutions of indole-3-acetic acid or abscisic acid in Tween-20, or 0.01% Tween-20 one week before the expected mid-véraison. The clusters were then harvested at mid-véraison and green, pink, and red berries counted (about 300 berries per treatment). Approximate test for equal proportions was used to identify differences in the distribution of the ripening classes between control and treated clusters, and significant differences were marked with asterisks ($p < 0.05$).
