# Supplementary material for: Timing of ripening initiation in grape berries and its relationship to seed content and pericarp auxin levels
Source: BMC Plant Biol. 2015 Feb 12;15:46. doi: 10.1186/s12870-015-0440-6 (PMC4340107; doi:10.1186/s12870-015-0440-6)
Supplement: Additional file 6: — Vitis gene IDs and sequences of the primers used in the qRT-PCR experiment. [file 12870_2015_440_MOESM6_ESM.pdf]

Additional file 6: Vitis gene IDs and sequences of the primers used in the qRT-PCR experiment.

Auxin response factor 4; *ARF4* (VIT_06s0004g03130)
F: GCTTGGAAGAAGCTCCAGTG
R: GCATACCAACAAGGCAAACA

Auxin response factor 6; *ARF6* (VIT_03s0167g00010)
F: AGCAAAAGGATGCGTACCTC
R: GAGGAACAGAGAAGCCTCCA

Auxin response factor 7; *ARF7* (VIT_13s0019g00110)
F: CACCATGGCAAGAATTGGTT
R: GCATCGCCAATTGCTAATCT

Auxin response factor 8; *ARF8* (VIT_04s0079g00160)
F: TTGGTGAGAGGAGGATTTGG
R: CCCAACCCTGATGTTGAAAG

Auxin/indole-3-acetic acid inducible protein 9; *Aux/IAA9* (VIT_13s0019g04380)
F: GCTAACAATTCTGGCCGATTC
R: TCACTCCCAAAAGCCAAAAC

9-cis-epoxycarotenoid dioxygenase; *NCED4* (VIT_02s0087g00930.t01)
F: CTTGATATTGTGGCCGCTGT
R: AATTCGAGCTTGCTTTCCAA

MYB transcription factor; *MYBA1* (VIT_02s0033g00410.t01)
F: GAGGTGAGGGTGATTTTCCA
R: AATGCAAGAACAACTTTTGAACT

TAR3; *TAR3* (VIT_218s0157g00090)
F: TGCTGCTGCACCAAATAAAA
R: TGAGCTTATGATCAACTGATTGTT

Gretchen Hagen3; *GH3-2* (VIT_207s0129g00660)
F: TCGATTCCAGAGTCCTCTCC
R: CGACAGATTGCCCTTTTGTT

Gretchen Hagen3; *GH3-1* (VIT_203s0091g00310.1)
F: TCCTCGACTCCAGAGTGGTC

R: AGTGGAACGGCCACTATTTG

YUCCA; *YUC1* (VIT_207s0104g01250)
F: AGGCTTGACTGGAGCTCGT
R: CCAAATTTTCTCATCCAAATCC

YUCCA; *YUC2* (VIT_207s0104g01260)
F: ATTTGAGGGTGGCAAATCAC
R: GGAAGTCCATCCTCGTTCAA

peptidyl-prolyl cis-trans isomerase (Endogenous control) (VIT_06s0004g06610)
F: TCCACTCTTGCCTTTTTGCT
R: ACGGATCAAAGCCATTTCTG
